# Identification and Single‐Cell Analysis of Viable Circulating Tumor Cells by a Mitochondrion‐Specific AIE Bioprobe

**DOI:** 10.1002/advs.201902760

**Published:** 2020-01-16

**Authors:** Bo Situ, Xinyi Ye, Qianwen Zhao, Liyao Mai, Yifang Huang, Siqi Wang, Jing Chen, Bo Li, Bairong He, Ye Zhang, Jianjun Zou, Ben Zhong Tang, Xinghua Pan, Lei Zheng

**Affiliations:** ^1^ Department of Laboratory Medicine Nanfang Hospital Southern Medical University Guangzhou 510515 China; ^2^ Guangdong Engineering and Technology Research Center for Rapid Diagnostic Biosensors Nanfang Hospital Southern Medical University Guangzhou 510515 China; ^3^ Department of Biochemistry and Molecular Biology School of Basic Medical Sciences Southern Medical University Guangzhou 510515 China; ^4^ Guangdong Provincial Key Laboratory of Single Cell Technology Application Guangzhou 510515 China; ^5^ Department of Oncology Guangzhou Chest Hospital Guangzhou 510515 China; ^6^ Guangdong Province Key Laboratory of Biomedical Engineering South China University of Technology Guangzhou 510006 China; ^7^ Department of Chemistry and Hong Kong Branch of Chinese National Engineering Research Center for Tissue Restoration and Reconstruction The Hong Kong University of Science & Technology Clear Water Bay Kowloon Hong Kong China; ^8^ HKUST‐Shenzhen Research Institute No. 9 Yuexing 1st RD, South Area, Hi‐tech Park, Nanshan Shenzhen 518057 China

**Keywords:** aggregation‐induced emission, circulating tumor cells, fluorescent probes, single‐cell analysis

## Abstract

Liquid biopsies of cancer via single‐cell molecular profiling of circulating tumor cells (CTCs) are hampered by the lack of ideal CTC markers. In this study, it is reported that TPN, a bioprobe with aggregation‐induced emission (AIE) activity is capable of distinguishing various tumor cells from blood leukocytes based on the difference in cell mitochondria. TPN is a cell‐permeant live‐cell stain that has little effect on cell viability and integrity, enabling single‐cell DNA/RNA analysis with improved efficiency compared with traditional antibody‐based methods. Using TPN labeling, CTCs and CTC cluster are detected in the blood from patients with lung or liver cancer. The capability of TPN to identify rare tumor cells in the malignant pleural effusion samples is also demonstrated. Furthermore, RNA sequencing of single lung CTC identified by TPN is successfully performed. The findings presented here provide an antibody‐free, low‐cost, and nondisruptive approach for detection and genomic characterization of viable tumor cells based on a mitochondria‐targeting AIE luminogen. It might serve as a new tool for monitoring of genomics dynamic of tumor and unraveling the mechanisms of tumor metastasis.

## Graphical abstract

A mitochondria‐targeting bioprobe with aggregation‐induced emission (AIE) activity for detecting rare tumor cells in the blood and pleural effusion from cancer patients is presented. The cell‐permeant, low toxic bioprobe enables genomic analysis of single tumor cells with improved performance, and can serve as a promising tool for liquid biopsy to obtain cancer genetics.

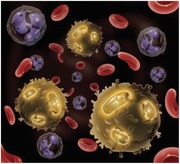

Metastasis is the hallmark of malignancies and the leading cause of cancer‐related deaths.^1^ The metastatic process depends on the spread of tumor cells through the bloodstream to form distant tumors.^2^ Circulating tumor cells (CTCs) are those tumor cells that are present in the blood of cancer patients and considered to be the “seeds” of tumor metastasis.^3^, ^4^ During the past decade, CTCs have attracted much attention as real‐time “liquid biopsy” biomarkers. Quantification of CTCs has been proven to be clinically valuable for prognosis prediction, treatment response assessing, and recurrence monitoring in various solid tumors.^5^, ^6^, ^7^, ^8^, ^9^, ^10^

Precision oncology aims to improve patient prognosis through molecular profiling of tumors. A particular advantage of CTCs compared with other cancer biomarkers is that they carry the whole genome from primary tumor.^8^ Molecular characterization of CTCs is thus more informative than CTCs counting, as it permits noninvasive monitoring of the changes in cancer genotypes for personalized therapy.^9^, ^10^ Although advances in sequencing technologies have enabled the detection of genome, transcriptome, and epigenome at single‐cell level,^11^ their applications in CTCs genotyping are still challenging. A major limitation is the high‐quality CTCs are often inaccessible for downstream analysis. As tumor cells are extremely rare (parts per billion of cells) in blood, CTCs must be enriched to increase their concentrations and identified by specific markers.^12^ Advanced technologies such as microfluidic‐based assays have allowed CTCs enrichment with minimal disruptions.^13^ However, during the procedures for CTCs identification, loss of cellular viability and integrity are usually unavoidable. For example, both the detection of cytokeratins (CKs), the “gold standard” protein markers of epithelial CTCs by immunofluorescence,^14^ and the aneuploidy of chromosomes by fluorescence in situ hybridization (FISH) require cell fixation and penetration to label the intracellular targets.^15^, ^16^ These procedures are prone to affect the quality of DNA and induce RNA degradation,^17^ which compromise their yields as single‐cell materials for downstream analysis in varying degrees. Detection of the cell‐surface epithelial proteins, for example, epithelial cell adhesion molecule (EpCAM) that express in many cancers has been reported to be a strategy to address the problem.^18^, ^19^, ^20^ However, their expression is lost after epithelial‐to‐mesenchymal transition (EMT), a common process by which CTCs lose their epithelial phenotype during cancer progression.^21^, ^22^ This leads to the limited sensitivity.

Mitochondria are essential organelles that are responsible for energy production in eukaryotes.^23^ Many studies have shown that the metabolic activities of mitochondria in tumor cells are distinctly different from that of normal cells and thus could serve as promising anticancer targets.^24^, ^25^ Our group has also reported that in cell line models, cancer cells emit stronger fluorescence than normal cells after mitochondria labeling, which may be due to the increased number of mitochondria with elevated mitochondrial membrane potential in the metabolically active tumor cells.^26^ Inspired by these findings, we envisaged that cellular mitochondria may serve as a potential marker for CTCs identification.

Here, we report a luminogen with aggregation‐induced emission (AIE) activity named TPN for noninvasive identification of tumor cells among blood cells. TPN can label mitochondria of multiple cancer cell lines with significantly higher fluorescence than that of white blood cells. Compared with traditional identification methods for CTCs marker, we show that TPN has little effect on cell quality and thus allows DNA or RNA to be analyzed on single‐cell level with improved performance. Using samples from cancer patients, we also demonstrate the capability of TPN to identify rare tumor cells in blood as well as malignant pleural effusion.

TPN consists of a prototypical AIE unit, tetraphenylethene (TPE), and a pyridinium group (**Figure** **1**a). It has an absorption maximum at 405 nm and an emission peak at 636 nm in DMSO.^27^ TPN is a cell‐permeant, live‐cell stain that is highly selective for the mitochondria. Its AIE attributes lead to the remarkable fluorescence “turn‐on” after binding to mitochondria.^28^ As leukocytes are the most abundant nucleated cells in blood, distinguishing of leukocytes and tumor cells is the most important criteria for a CTCs marker. To test our hypothesis, leukocytes that are isolated from blood and seven different tumor cell lines (H1975, A549, SMMC‐7721, HepG2, HT‐29, MCF‐7, and HeLa) were incubated with TPN respectively under the same staining condition. We observed that most of the human leukocytes show weak emission while all the seven tumor cell lines emit significantly brighter fluorescence (Figure 1b). We next mixed a large number of H1975 cells (≈5000) into leukocytes to measure their fluorescent intensity by flow cytometry. In line with the imaging results, quantitative analysis shows that the mean fluorescent signal of H1975 cells was 8.2 times higher than that of leukocytes (Figure 1c,d). Further detection of multiple cancer cell types shows that they emit 3.7–12.9 times (A549 4.1, MDA‐MB‐231 4.7, MCF‐7 12.9, HT‐29 4.4, HeLa 6.4, HepG2 5.4, SMMC‐7721 3.7) brighter emission than leukocytes (Figure 1e; Figure S1, Supporting Information). This difference in fluorescence suggests that TPN has the potential to distinguish tumor cells among leukocytes in a phenotype‐independent manner.

**Figure 1:**
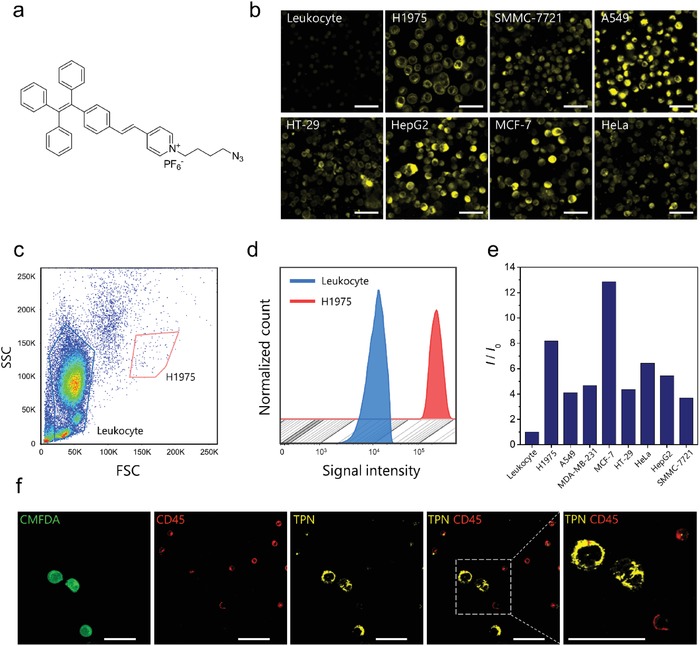
The structure and ability of TPN to identify various tumor cells within leukocytes. a) Chemical structure of TPN. b) Fluorescence images of different types of cells and blood leukocytes stained with TPN (2 × 10^−6^ m) for 10 min. c) Flow cytometry profiles and d) signal intensity leukocytes and H1975 cells after labeled with TPN. e) Fluorescent intensity of TPN‐stained leukocytes and various cancer cell lines measured by flow cytometry. f) Fluorescence images of leukocytes spiked with a low number of H1975 cells prelabeled by CellTracker CMFDA, followed by staining with TPN and anti‐CD45. Excitation wavelength: 488 nm (for CMFDA), 405 nm (for TPN), and 594 nm (for anti‐CD45). Scale bars, 50 µm.

As flow cytometry does not possess the sensitivity to analyze rare cells in blood, we next spiked a small number of tumor cells in leukocytes to mimic the real samples. A serial of known numbers (5–110) of lung cancer cells (H1975) were prelabeled with 5‐chloromethylfluorescein diacetate (CMFDA), a green‐emissive dye, followed by spiking into leukocytes and costained with TPN and anti‐CD45 antibodies (leukocyte marker). Cells that emitted bright yellow fluorescence were considered as tumor cells and verified by the existence of CMFDA emission. As shown in Figure 1f, most of the leukocytes (CMFDA^−^/CD45^+^) were weakly emissive. In contrast, intense yellow fluorescence could be found from the reticulum‐like mitochondria within H1975 cells (CMFDA^+^/CD45^−^), which can be well distinguished from the leukocytes. Based on the marked difference in fluorescence, we identified 94.0 ± 3.9% (mean ± standard deviation (SD)) of the spiked H1975 cells. In addition, high recovery (97.2 ± 3.1%, mean ± SD) was also achieved using liver cancer cells (HepG2) as model (Figure S2 and Table S1, Supporting Information). These results indicate that TPN is capable of labeling tumor cells with high efficiency and selectivity. We also used MitoTracker Green (MTG), a well‐known commercial mitochondrial dye for comparison. Analysis by flow cytometry shows that the mean fluorescent signal of A549 cells is just 1.2 times higher than that of leukocytes (Figure S3, Supporting Information), which is significantly lower than that of the TPN (4.1 times). We observed that tumor cells spiked into leukocytes exhibit faint fluorescence after MTG labeling. Compared with TPN staining, the difference in MTG signal between leukocytes and tumor cells is too small to distinguish (Figure S4, Supporting Information). Since the working concentration of MTG in this experiment is relatively low (200 × 10^−9^ m, maximum concentration in the recommended range), the accompanying low dynamic range may attribute to the indistinctive difference in cellular fluorescence. Further increasing the concentration of these probes may result in the aggregation‐caused quenching (ACQ) effect and nonspecific binding.^29^ In contrast, benefitting from the unique AIE property,^30^, ^31^, ^32^, ^33^, ^34^ higher working concentration (2 × 10^−6^ m) of TPN was used and the emission of TPN could be intensified along with the increased accumulation of dye in mitochondria of tumor cells. This could be important to its better selectivity to tumor cells.

We next sought to evaluate the effect of TPN labeling on downstream analysis. Propidium iodide (PI), a stain for dead cell, was used to examine cell viability after TPN labeling (Figure S5, Supporting Information). We observed that most cells from various cell lines remained viable after TPN and PI staining (H1975 98.9%, A549 97.0%, SMMC‐7721 100%, HepG2 95.8%, MDA‐MB‐231 92.8%, HT‐29 98.00%, Figure S6, Supporting Information), indicative of the low toxicity of TPN.

To further test whether TPN would affect molecular analysis at the single‐cell level, we evaluated the performance of TPN labeling in downstream analysis and used traditional CKs marker that were stained by immunofluorescence as a comparison (**Figure** **2**a). Single A549 cells that were labeled with TPN or immunofluorescence were isolated (Figure 2b) and lysed to release intracellular nucleic acids, followed by assessing the single‐cell genomic (DNA) or transcriptomics (RNA) information. Due to the low abundance of materials within one cell (picograms), successful amplification of nucleic acid from individual cells to obtain micrograms of DNA were crucial for downstream analysis. For single‐cell genomic analysis, we first used multiple displacement amplification (MDA) for whole‐genome amplification (WGA) of individual single cells isolated by manual picking. MDA product was then submitted to PCR reactions for ten different genetic loci (Table S2, Supporting Information) followed by gel electrophoresis to evaluate the genomic coverage. As shown in the electropherograms (Figure 2c), all the ten genetic loci in five single A549 cells labeled with TPN were detectable, indicating the successful single‐cell WGA with high genomic coverage. The high‐quality of MDA product also allows Sanger sequencing to be successfully performed. From the sequencing data (Figure 2e) we may found the characteristic Kirsten rat sarcoma (KRAS) gene mutation (p.G12S c.34G>A) within single A549 cell.^35^ In contrast, only 3 out of 5 single cells that were labeled by immunofluorescence presented 1–2 expected bands in ten genetic loci (Figure 2c,d)) and the downstream analysis by Sanger sequencing was unable to report a specific result (data not show), suggesting the low DNA yield. We also used multiple annealing and looping based amplification cycles (MALBAC), another WGA method for testing. Similarly, MALBAC product from single cells labeled with TPN covered significantly wider distribution across the genome than those by immunofluorescence staining (Figure S7, Supporting Information).

**Figure 2:**
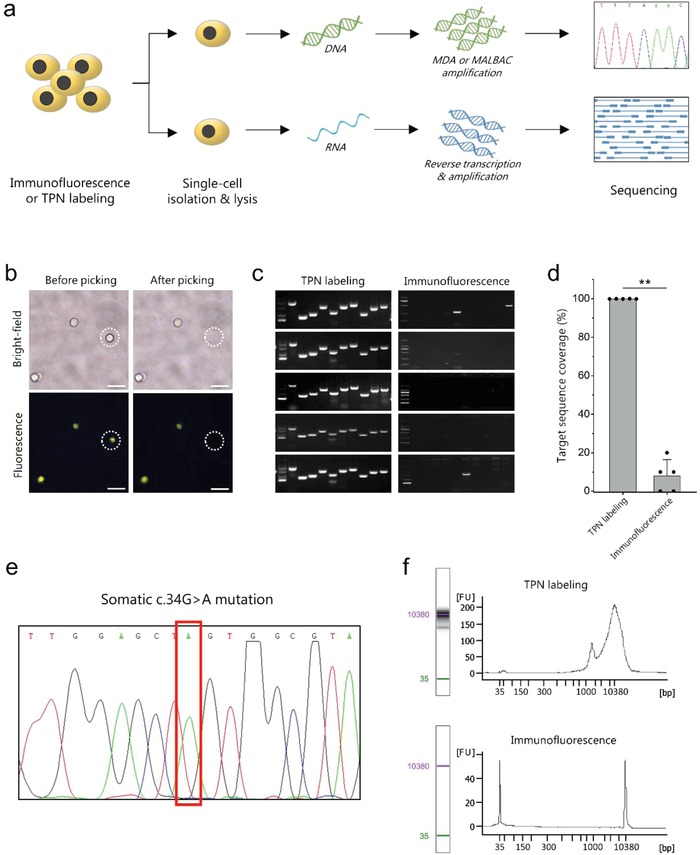
Evaluation of TPN and immunofluorescence labeling on downstream single‐cell analysis. a) Schematic illustration of the experimental workflow. b) Representative images of single labeled cell before and after successful isolation. c) Electropherograms and d) mean genomic coverages of whole‐genome amplification products by MDA from TPN or CKs labeled single A549 cells assessed by ten genomic loci. Experiments for each labeling methods were repeated using five single cells. Values are the mean of five replicates and error bars represent the standard deviation (SD). Mann–Whitney U test, **P < 0.01. e) KRAS mutation revealed by Sanger sequencing of the MDA product from single TPN‐labeled A549 cell. f) Electropherograms and corresponding signal distribution of the cDNA products reverse transcribed from RNA within single cell labeled by TPN or immunofluorescence. Scale bars, 20 µm.

In addition, the quality of RNA from single cells was also evaluated. RNA from single cells that were labeled with either of TPN or CKs were lysed, reverse transcribed to cDNA, and amplified. Amplicons were then detected by the Agilent 2100 Bioanalyzer. From the electropherogram of the TPN‐labeled cell, we observed the cDNA signal peak ranging from around 200–2000 bp at a concentration of 20 034.1 pg µL^−1^ (Figure 2f), indicative of the high quality of cell‐derived RNA that is sufficient for downstream analysis. In sharp contrast, negligible amplified product (8.2 pg µL^−1^) can be detected from the CKs‐labeled cell (Figure 2f), suggesting that immunofluorescence staining brought about a negative impact on single‐cell RNA detection. The above data indicates that labeling of TPN has little effects on cellular downstream analysis even when working with one cell. This is because TPN is a fluorescent “light up” probe that can easily diffuse across the cell membrane and accumulate in the mitochondria within living cell as we reported previously.^28^ The labeling procedures are simple with little interference to cell biology. For CKs labeling, serial disruptive steps including cell fixation and permeabilization are required.^36^ These procedures can induce nucleic acids cross‐linking and fragmentation,^37^ which greatly inhibit the activity of polymerase or reverse transcriptase and compromise the amplification efficiency.

We then evaluated the performance of TPN with clinical samples. Whole blood samples were collected from patients with advanced lung cancer (*n* = 68) and liver cancer (*n* = 22) (Tables S3 and S4, Supporting Information). CTCs were first enriched by a spiral microfluidic‐based automated cell retrieval system (ClearCell FX),^38^, ^39^ followed by staining with TPN, CD45 (leukocyte marker) antibodies, and Hoechst (nuclear dye). We also tested the blood from healthy volunteers (*n* = 12). Nucleated cell emits strong yellow fluorescence, absent staining for CD45 (TPN^+^/CD45^−^/Hoechst^+^) with a high nuclear/cytoplasm ratio but without a segmented nucleus (characteristics of the granulocytes) was enumerated as a putative CTC. Based on this criteria, CTCs were detected in 53 of 68 patients (77.9%) with lung cancer (**Figure** **3**a), ranging from 1 to 57 CTCs per 5 mL of whole blood (4.2 ± 7.4 CTCs). Of the 22 patients with liver cancer, 11 (50%) had detectable CTCs ranging from 1 to 8 per 5 mL of whole blood (1.2 ± 1.9 CTCs). As CTCs were enriched by the size‐based ClearCell FX system, the lower detection rate in blood of liver cancer may be due to the relatively smaller size of liver CTCs.^40^ In the healthy donors group, only 1 out of 12 volunteers has 2 putative cells, which is significantly lower than the cancer patients groups. The two putative cells may be metabolically active immature leukocytes that are released from the bone marrow. Figure 3b shows the representative images of putative cancer cells that are Hoechst positive and CD45 negative with brighter emission of TPN from the blood of patients. Leucocytes show a low background of TPN fluorescence with CD45 positive. It is noteworthy that we have also found a CTC cluster from the blood of a lung cancer patient (Figure 3c). The CTC cluster consisting of three aggregating CTCs with irregular size and shape, and was adhered by a typical neutrophil characterized by its lobulated shape of nucleus.^41^ We observed that the CTC cluster emits significantly brighter yellow fluorescence from cytoplasm than that of the adjacent neutrophil, which can be ascribed to the metabolically active tumor cells with more mitochondria stained by TPN. These results suggest that TPN is capable of labeling single CTC and CTC cluster in blood, and can serve as a reliable CTC marker for distinguishing cancer from healthy controls. We also examined the samples of malignant pleural effusion, a common but serious condition caused by metastasized tumor cells in pleura.^42^ Two malignant pleural effusion samples that had been diagnosed by cytology were used to test. As shown in Figure 3d, we detected a large number of TPN^+^CD45^−^ cells (>1000 per mL) in both specimens. In contrast, no putative cells were found in three pleural effusion samples caused by benign diseases (Figure S8 and Table S5, Supporting Information), demonstrating the high potential of TPN as a new tool for malignant pleural effusion diagnosis.

**Figure 3:**
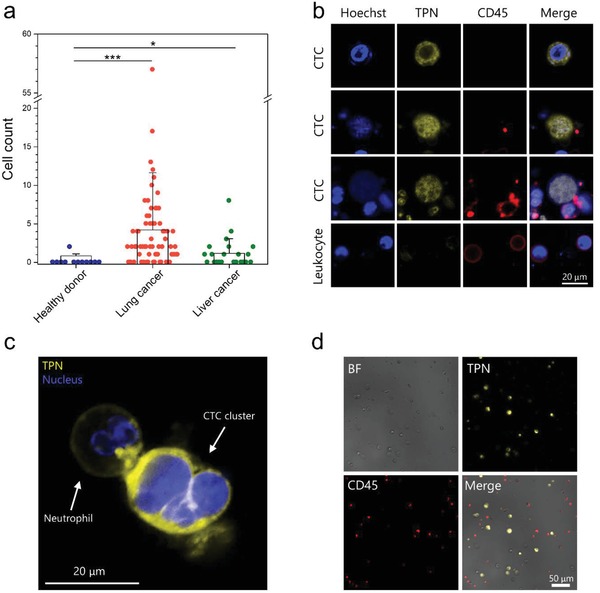
Rare tumor cells detected in blood and pleural effusion from cancer patients. a) Counts of candidate cells in healthy donors, patients with lung cancer, and patients with liver cancer. Independent *t*‐test, *P < 0.05, ***P < 0.001. b) Representative fluorescence images of tumor cells and leukocytes from the blood of cancer patients. c) Fluorescence image of a CTC cluster adhere to a neutrophil in blood. d) Representative bright‐field and fluorescence images of cells from the pleural effusion of a lung cancer patient.

The superior performance of TPN prompted us to test its feasibility in single CTC RNA sequencing that relies heavily on cell quality. We micromanipulated single TPN^+^/CD45^−^/Hoechst^+^ cell from the blood of a lung cancer patient for next‐generation RNA sequencing. The isolated cell has sufficient RNA to generate high‐quantity amplified cDNA (Figure S9, Supporting Information) for library construction and transcriptome sequencing. The sequencing data got scores of higher than 32 assessed by Phred quality scoring (Figure S10, Supporting Information), indicative of its reliability. The sequencing data show that among the most abundantly expressed transcripts, we may find genes of MALAT1, GSTO1, CTTN, THBS1, and SPARC that have been reported to be correlated with tumorigenesis,^43^, ^44^, ^45^, ^46^, ^47^ and absent expression of hematopoietic cell markers^48^ including CD45, CD14, CD56, CD4, and CD8 (Figure S11, Supporting Information). We further use complete‐linkage clustering to analyze the sequencing data from the isolated cell, 55 lung cancer samples, and 12 normal tissues datasets from the Cancer Genome Atlas (TCGA) network (Table S6, Supporting Information). As shown in **Figure** **4**, the gene expression pattern of the isolated cell was strongly clustered into the lung cancer group, which indicated the high probability of malignancy of the cell.

**Figure 4:**
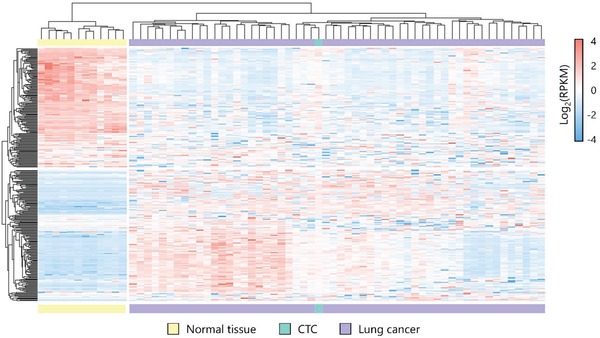
Heat map illustrating the complete‐linkage clustering analysis of RNA‐sequencing data from lung cancer samples (purple, *n* = 55), normal tissue samples (yellow, *n* = 12), and single CTC (green) showing the top 100 differentially expressed genes.

In summary, we have developed a mitochondria‐targeting AIE bioprobe (TPN) for detection of rare tumor cells among normal cells. After incubation of TPN, tumor cells can be well‐distinguished from white blood cells with high‐contrast because they exhibited stronger TPN fluorescence than human leukocytes. This may due to the metabolically active tumor cells have more mitochondria with elevated mitochondrial membrane potential^49^, ^50^ than mature leukocytes in order to maintain high energy production for rapid cell division. We further demonstrate the capability of TPN to identify tumor cells in peripheral blood and malignant pleural effusion samples from lung and liver cancer patients. Importantly, unlike conventional CTCs identification methods, the antibody‐free, live‐cell labeling assay is extremely simple, low‐cost, and has little effect on cell viability and integrity, which allows high‐quality analysis of tumor cell on single‐cell level. Combined with the antigen–independent, microfluidic‐based device for CTCs enrichment, TPN can enable identification of CTCs in suspension. This immobilization‐free workflow facilitates the convenient manipulation of single cell for genomic analysis. In addition, cell viability can be maximally maintained and thus well suited for CTCs culturing, functional analysis, and developing of xenotransplantation animal models to gain insights into CTCs properties. Considering the well performance of TPN on various tumor cell lines, TPN labeling offers potential for the identification of rare cells of other types of cancer. Furthermore, the detection workflow can also be potentially extended to detect rare tumor cells in other body fluids such as urine, cerebrospinal fluid, as well as peritoneal effusion. One major limitation of this method is that the identification process has high requirement on cell viability. Sample needs to be processed in a short time and tumor cells should be enriched using platform with minimal disruptions to cells in order to avoid nonspecific diffusion of probe into leukocytes with impaired membranes, as well as the decreased uptake of TPN in tumor cells with low activity. This could be addressed in future by improving the properties of probe.

## Experimental Section

Experimental details can be found in the Supporting Information.

## Conflict of Interest

The authors declare no conflict of interest.

## Supporting information

Supporting Information
