# Supplementary material for: Identification and Single‐Cell Analysis of Viable Circulating Tumor Cells by a Mitochondrion‐Specific AIE Bioprobe
Source: Adv Sci (Weinh). 2020 Jan 16;7(4):1902760. doi: 10.1002/advs.201902760 (PMC7029725; doi:10.1002/advs.201902760)
Supplement: Supplementary file 1 — Supporting Information [file ADVS-7-1902760-s001.pdf]

Copyright WILEY-VCH Verlag GmbH & Co. KGaA, 69469 Weinheim, Germany, 2020.

ADVANCED SCIENCE
Open Access

Supporting Information

for *Adv. Sci.*, DOI: 10.1002/advs.201902760

Identification and Single-Cell Analysis of Viable Circulating Tumor Cells by a Mitochondrion-Specific AIE Bioprobe

*Bo Situ, Xinyi Ye, Qianwen Zhao, Liyao Mai, Yifang Huang, Siqi Wang, Jing Chen, Bo Li, Bairong He, Ye Zhang, Jianjun Zou, Ben Zhong Tang, Xinghua Pan, and Lei Zheng**

Copyright WILEY-VCH Verlag GmbH & Co. KGaA, 69469 Weinheim, Germany, 2019.

Supporting Information

Identification and Single-Cell Analysis of Viable Circulating Tumor Cells by a Mitochondrion-Specific AIE Bioprobe

*Bo Situ[†], Xinyi Ye[†], Qianwen Zhao[†], Liyao Mai, Yifang Huang, Siqi Wang, Jing Chen, Bo Li, Bairong He, Ye Zhang, Jianjun Zou, Ben Zhong Tang, Xinghua Pan, and Lei Zheng**

Dr. B. Situ, X. Ye, Q. Zhao, Y. Huang, Dr. J. Chen, Dr. B. Li, Dr. B. He, Y. Zhang, Prof. L. Zheng
Department of Laboratory Medicine, Nanfang Hospital, Southern Medical University, Guangzhou, 510515, China
E-mail: nfyyzhenglei@smu.edu.cn
Dr. B. Situ, X. Ye, Q. Zhao, Y. Huang, Dr. J. Chen, Dr. B. Li, Dr. B. He, Y. Zhang, Prof. L. Zheng
Guangdong Engineering and Technology Research Center for Rapid Diagnostic Biosensors, Nanfang Hospital, Southern Medical University, Guangzhou 510515, China
L. Mai, S. Wang, Prof. X. Pan
Department of Biochemistry and Molecular Biology, School of Basic Medical Sciences, Southern Medical University, Guangzhou, 510515, China
L. Mai, S. Wang, Prof. X. Pan
Guangdong Provincial Key Laboratory of Single Cell Technology Application, Guangzhou, 510515, China
Dr. J. Zou
Department of Oncology, Guangzhou Chest Hospital, Guangzhou, 510515, China
Prof. B. Z. Tang
Guangdong Province Key laboratory of Biomedical Engineering, South China University of Technology, Guangzhou, 510006, China
Prof. B. Z. Tang
Department of Chemistry and Hong Kong Branch of Chinese National Engineering Research Center for Tissue Restoration and Reconstruction, The Hong Kong University of Science & Technology, Clear Water Bay, Kowloon, Hong Kong, China
HKUST-Shenzhen Research Institute, No. 9 Yuexing 1st RD, South Area, Hi-tech Park, Nanshan, Shenzhen, 518057, China
[†] These authors contributed equally to this work.

Experimental Section

Reagents and Materials: Fetal bovine serum (FBS), Dulbecco's modified eagle medium (DMEM), penicillin and streptomycin were purchased from Invitrogen. RBC lysis buffer, PE-conjugated anti-CD45 was obtained from BD Biosciences. FITC-conjugated anti-CKs was purchased from Abcam. 5-chloromethylfluorescein diacetate (CellTracker Green, CMFDA), MitoTracker Green (MTG), Dimethyl sulfoxide (DMSO), propidium iodide (PI), Hoechst 33342, Triton X-100 were purchased from Beyotime. REPLI-g Single Cell Kit was obtained from Qiagen. Single Cell Whole Genome Amplification Kit was obtained from Yikon Genomics. All other reagents used in this study were obtained from Sigma-Aldrich and used as received without further purifcation.

Synthesis of TPN: 1-(4-Azidepropyl)-4-methylpyridinium hexafluorophosphate (TPN) was synthesized according to the previous study.[1]

Cell Culturing: SMMC-7721, HepG2, HT-29, MCF-7, and HeLa cells were cultured in DMEM while H1975 and A549 cells were cultured in RPIM-1640 containing 10% FBS and 1% Penicillin-Streptomycin at 37 °C with 5% CO_2.

Leukocyte Preparation: 1 ml of whole blood was obtained for a healthy donor. Leukocyte were suspended in PBS after removing red blood cells using a lysis buffer (BD Bioscience).

Cell imaging: Same amount (10^4) of different cultured cancer cells and leukocyte were stained with 2×10^{-6} M of TPN for 10 min, followed by washing three times with phosphate buffered saline (PBS) before observation. Cells were then imaged by a confocal microscope (Zeiss LSM 880).

Flow Cytometry Analysis: Leukocyte and various cancer cells were first analysed by FACS Calibur flow cytometer (BD LSRFortessa) respectively to set their gates. 5000 tumor cells were spiked into 1×10^6 leukocytes and incubated with anti-CD45 antibodies (1:200) for 30 min at room temperature, followed by staining with 2×10^{-6} M of TPN for 10 min or 2×10^{-7} M

of MTG for 30 min. Fluorescence intensities from the gates of leukocyte and tumor cells were recorded. Data were analyzed with FlowJo software (FlowJo, USA).

Recovery Testing: Cultured cells were labeled with CellTracker Green CMFDA of 50×10^{-9} M for 15 min. A serial of known numbers (5-110) of pre-labeled cells were then spiked into 3×10^{4} leukocytes, followed by incubation with CD45 antibodies (1:200) for 30 min at room temperature and then stained with 2×10^{-6} M of TPN for 10 min. Cells were washed three times with PBS before imaging. The cells emitted bright yellow fluorescence were counted under a fluorescence microscope (Olympus CKX41).

Cell Viability Evaluation: Cultured A549 cells were pre-labeled with CellTracker Green CMFDA (50×10^{-9}) M for 15 min and diluted to ~2000 cells/ml. Cells were then stained with 2×10^{-6} M TPN for 10 min and the excess dye was removed by washing 3 times with PBS. Labeled cells were then incubated in RPIM-1640 containing 10% FBS at 37 °C with 5% CO_2 for 2 h. Before counting under a microscope (Olympus CKX41), cells were stained with 1.5×10^{6} M propidium iodide (PI) for 5 min. Total cells and PI positive cells counts were recorded to calculate the cell viability.

Cell Immunofluorescence Staining: A549 cell suspension was fixed by 4% paraformaldehyde and permeabilized with 0.2% Triton X-100 in PBS for 10 min. 4% bovine serum was then added to block the nonspecific binding sites for 30 min before staining with the FITC-conjugated antibodies to CKs (1:200) for 1 h.

Isolation of Single Cell: Cell was manually picked with a glass capillary. To confirm the successful separation of single cells without losing, each isolated cell was observed under a microscope after manual picking to make sure that it has been removed. After single cell was transferred into a PCR tube containing reaction buffer, the glass capillary was rinsed in a drop of PBS on a slide and observed under a microscope to confirm the absence of the cell.

Single-cell Whole Genome Amplification (WGA): Single labeled cells were isolated by manual manipulation into PCR tubes under a microscope. Individual single cell was amplified by

MDA REPLI-g Single Cell Kit (Qiagen) or MALBAC Single Cell Whole Genome Amplification Kit (Yikon Genomics) according to their protocols. WGA quality was assessed by agarose gel electrophoresis of the products of PCR reactions for 10 different gene loci.

KRAS Mutation Sequencing: KRAS gene was amplified by PCR from MDA products of single TPN-labeled cell. The amplicons were purified and sequenced directly from both sides on an ABI3730xl automated capillary sequencer (Applied Biosystems, USA).

Detecting CTCs from Blood Samples: 5 ml blood samples collected in EDTA-contained vacutainer tubes were obtained from healthy volunteers and cancer patients with informed consent and approval by the ethics board of Nanfang Hospital. Blood samples were processed within 4 hours after collection. The erythrocytes were removed by lysing for 10 min at room temperature and the remaining nucleated cells were enriched by a ClearCell FX device. Enriched cells were then incubated with anti-CD45 (1:200), Hoechst 33342 (1:500) for 45 min, and 2×10^{-6} M TPN for 10 min.

Detecting Rare Tumor Cells from Pleural Effusion: 10 ml of pleural effusion was first centrifuged at 500 g for 10 min to remove cell debris. Resuspended cells were then enriched in the ClearCell FX device. Enriched cells were then incubated with anti-CD45 (1:200) for 45 min, followed by incubating with 2×10^{-6} M TPN for 10 min.

Single-cell RNA Sequencing: Single CTC was manually isolated into a tube with lysis buffer containing 1 μl dNTP mixture (Fermentas), 1 μl Oligo dT primer, 1.9 μl 0.2% Triton X-100, and 0.1 μl RNase Inhibitor (Takara). Single-cell transcriptome amplification was then conducted following the Smart-Seq2 protocol.[2] The amplified cDNA products were purified with 1x Agencourt Ampure XP beads (Beckman) and then assessed by an Agilent 2100 bioanalyzer. Library was then constructed using the Nextera XT DNA sample preparation kit (Illumina), and sequenced by an Illumina HiSeqX10 sequencing platform with 150 bp pair-end reads.

Data Processing: The adapter sequences, TSO sequences, low quality bases, and "N" bases was filtered out by Cutadapt (v1.12) and Trimmomatic (v0.38). Data were then aligned to reference genome by FastQC (v0.11.5) and the quality was assessed by Phred quality scoring with RSeQC. For data analysis, RNA sequences from the single cell were aligned to the known human transcriptome (hg38) using TopHat (32). After .bam files sorting (samtools), HTseq-count (v0.11.1) was used to count the aligned sequencing reads number and calculate the RPKM expression matrix. A training set was first constructed using data from 12 normal tissue and 55 lung cancer tissue from TCGA database with matching background of the patient. 1870 differentially expressed genes in the training set were selected by edgeR (foldChange=2 and padj=0.01) and batch effect was excluded by limma R Packages. The top 100 differentially expressed genes were selected for clustering analysis with the single-cell RNA sequencing data.

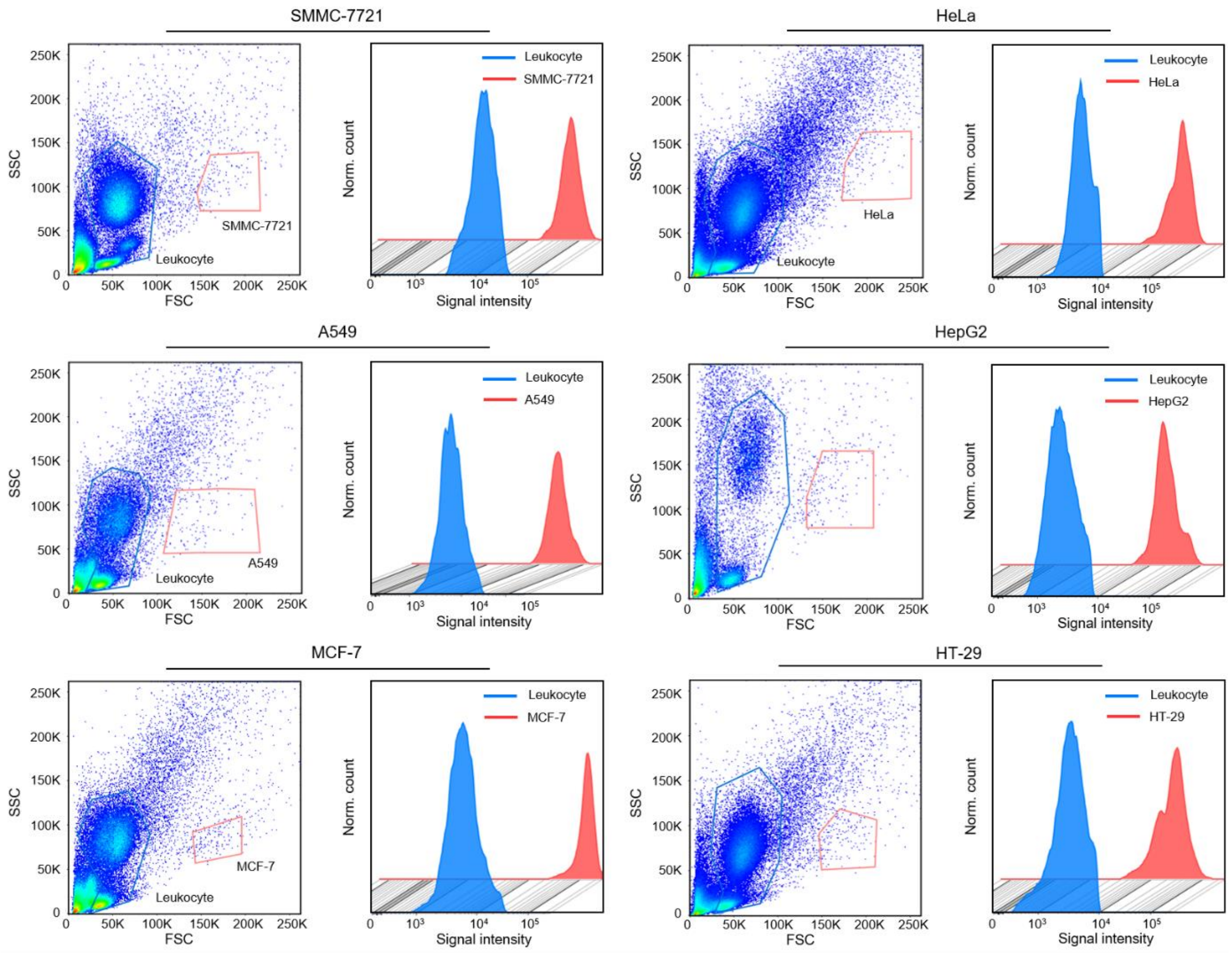

Figure S1. Flow cytometry profiles and signal intensity of cell mixtures containing leukocytes and various cancer cells (SMMC-7721, HeLa, A549, HepG2, MCF-7, HT-29) after labeled with TPN.

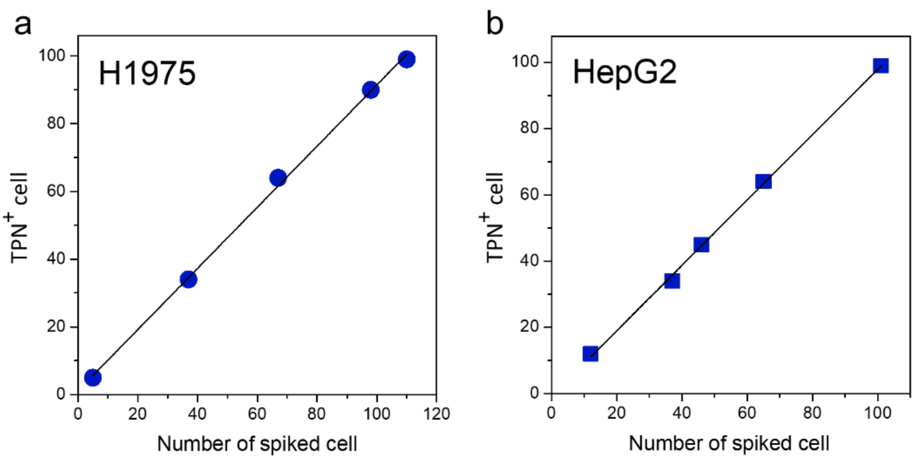

Figure S2. Recovery testing by spiking a known number of H1975 and HepG2 cancer cells into leukocytes.

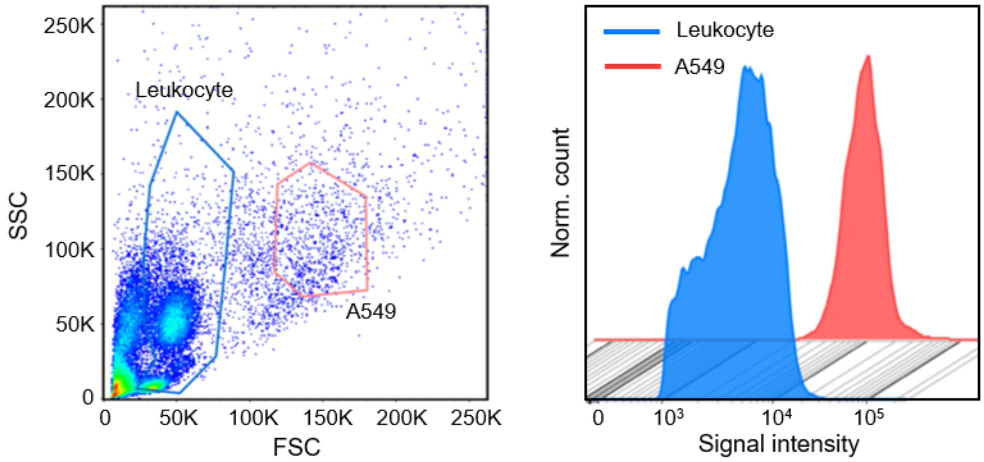

Figure S3. Flow cytometry profiles and signal intensity of cell mixtures containing leukocytes and A549 cells stained with 200 nM MTG for 30 mins.

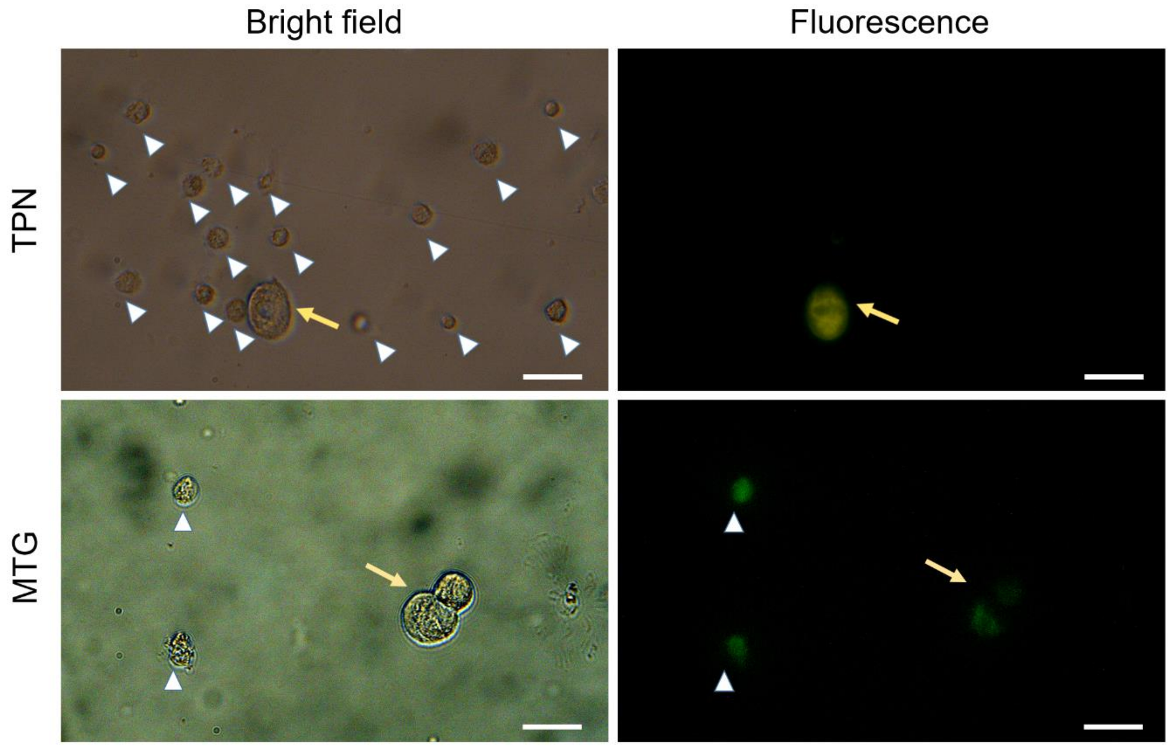

Figure S4. Bright field and fluorescent images of cell mixtures containing A549 and leukocytes co-stained with TPN or MTG. Yellow arrows indicate the cancer cells and the white arrowheads show the leukocytes. Scale bars, 20 μm.

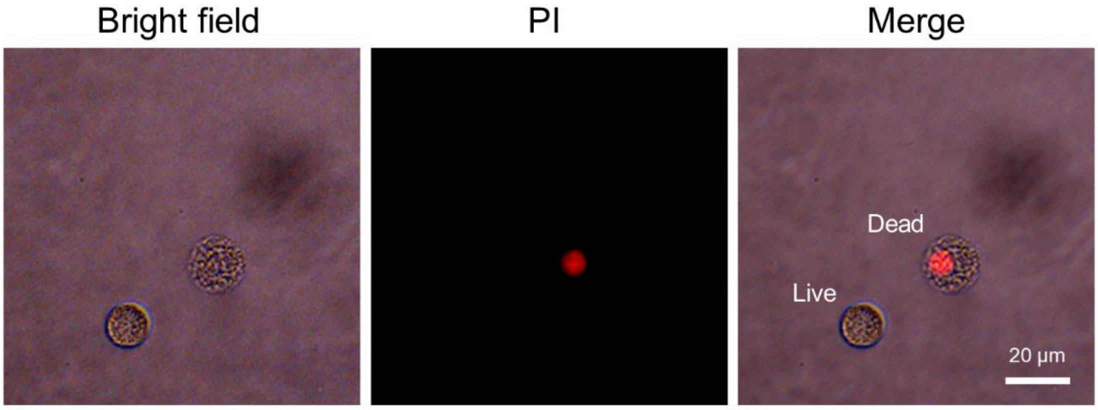

Figure S5. Evaluation of cell viability after staining of TPN by Propidium Iodide (PI) staining. Cell viability was determined by manually counting of the dead cells (PI+) and total cells. Excitation wavelength: 495–530 nm.

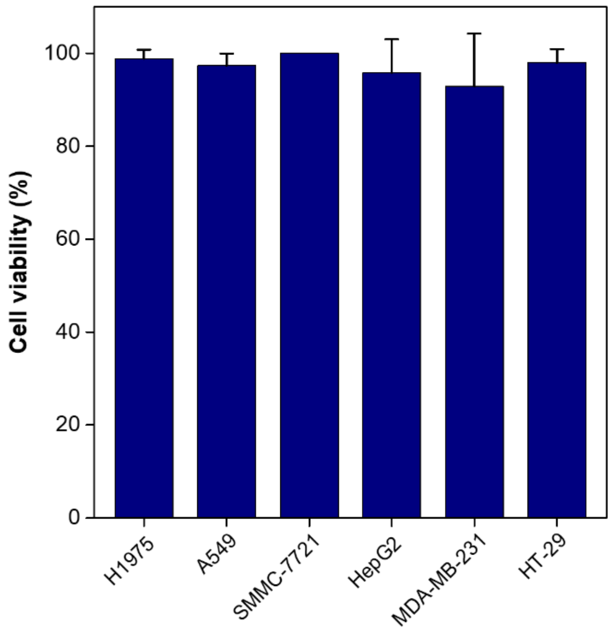

Figure S6. Cell viability of different types of cancer cells after labeling with TPN evaluated by PI staining. Values are the mean of three replicates and error bars represent the standard deviation (SD).

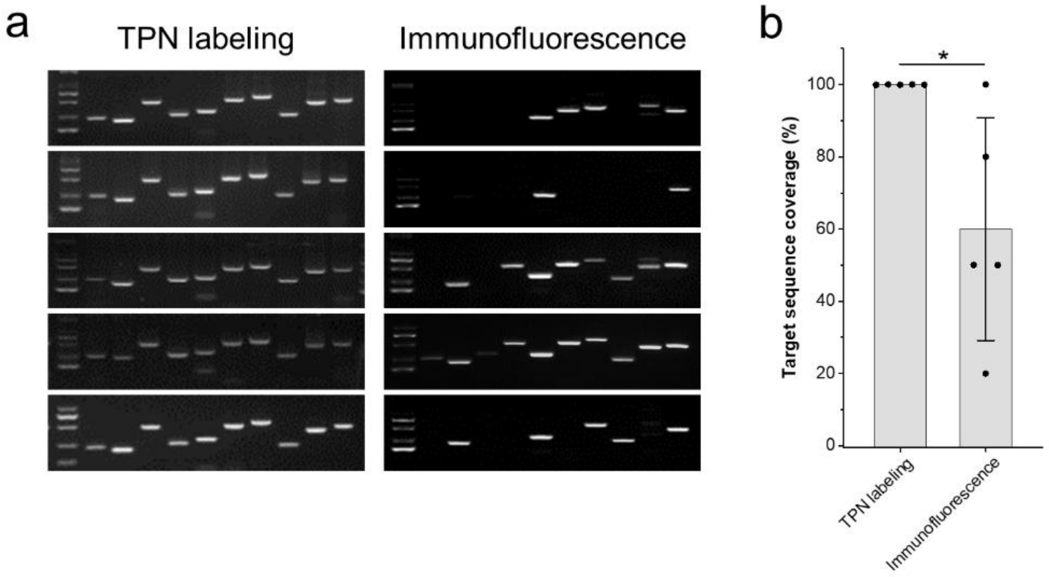

Figure S7. a) Electropherograms and b) mean genomic coverages of whole-genome amplification product by MALBAC from TPN or CKs labeled single A549 cells (five individual cell for each method) assessed by 10 genomic loci. Mann-Whitney U test was used for statistical analysis, *$P < 0.05$. Values are the mean of 5 replicates and error bars represent the standard deviation (SD).

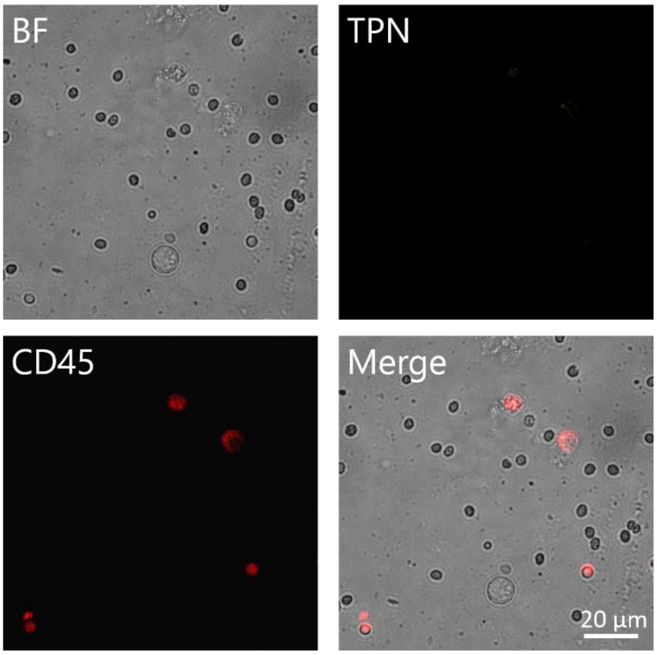

Figure S8. Representative bright field and fluorescent images of cells from the pleural effusion of a patient (PE5) with benign disease (congestive heart failure).

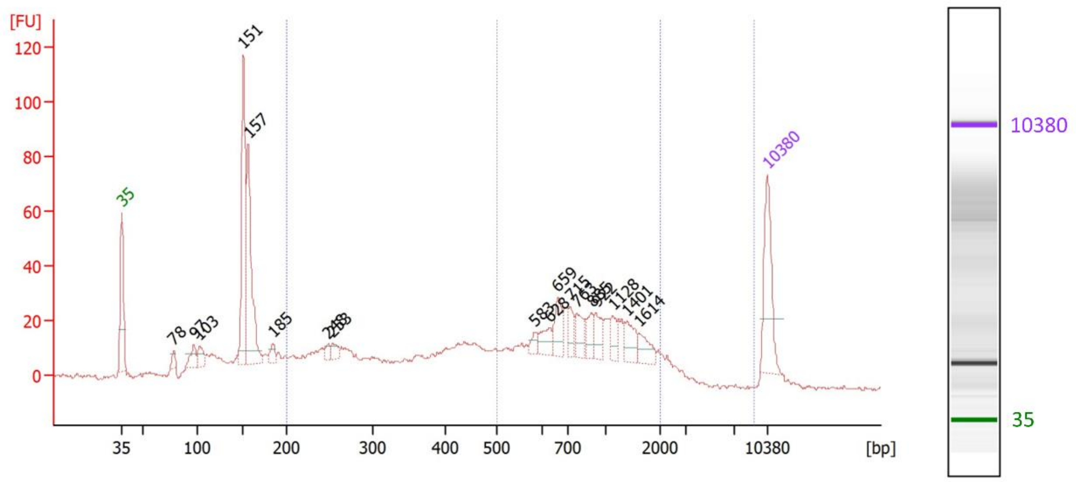

Figure S9. Electropherogram and corresponding signal distribution of the cDNA products reverse transcribed from RNA within single CTC identified by TPN. A concentration of 1264.5 pg/µl was determined at ranging from 200 bp to 2000 bp.

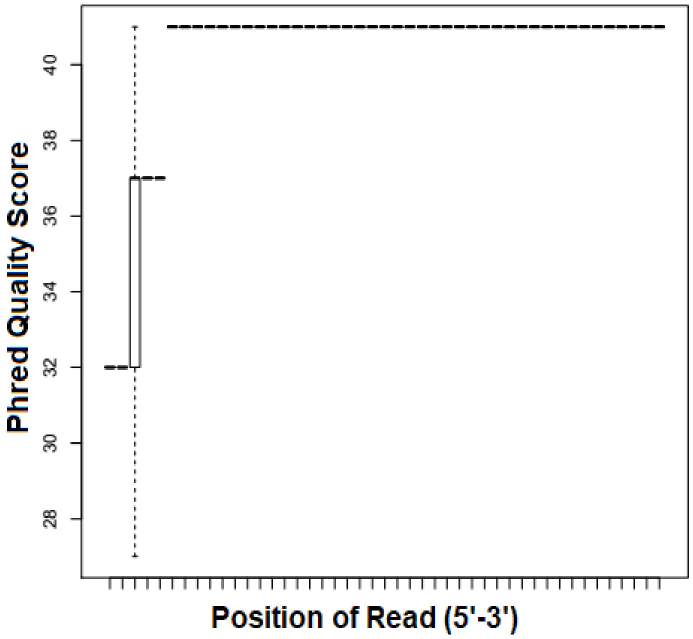

Figure S10. Then quality of reads assessed by Phred Scoring.

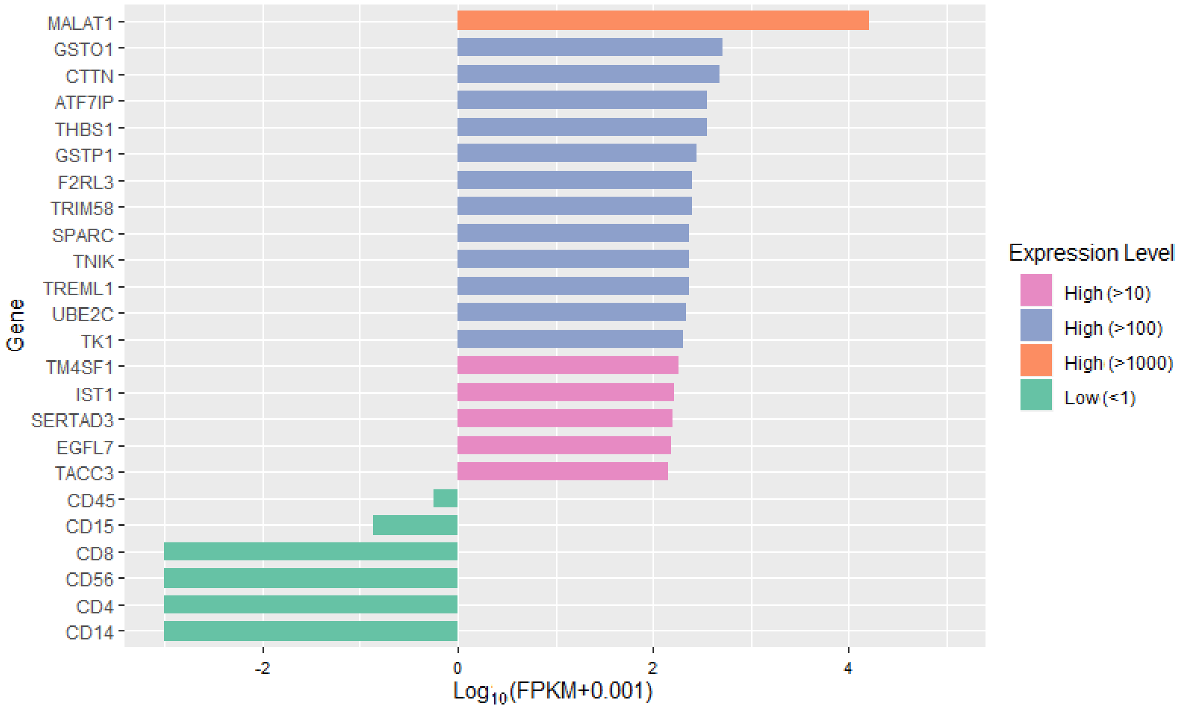

Figure S11. Representative high or low expression of genes of the single isolated cell.

Table S1. Counts of TPN+ cells after spiking different numbers of H1975 and HepG2 cells in the buffer containing leukocytes.

	H1975		HepG2	
	# of spiked cell	# of identified cell	# of spiked cell	# of identified cell
Test 1	5	5	12	12
Test 2	37	34	37	34
Test 3	67	64	46	45
Test 4	98	90	65	64
Test 5	110	99	101	99

Table S2. Primer sequences for ten gene loci.

Number	Sequences of primer pairs (5'-3')	Size (bp)
1	TTATAAGGCCTGCTGAAAATGACTG	211
	TCATGAAAATGGTCAGAGAAACC	
2	GGGAAAAATATGACAAAGAAAGC	250
	CTGAGATCAGCCAAATTCAGTT	
3	AGATCTACTGTTTTCCTTTACTTACTACACC	184
	AATCAGTGGAAAAATAGCCTCAATTCT	
4	GCAATGGATGATTTGATGCTGTCC	308
	GCCAGGCATTGAAGTCTCATGGAA	
5	CAACTCTGTCTCCTTCCTCTTC	533
	GCACATCTCATGGGGTTATAGG	
6	ACAGGTCTCCCCAAGGC	644
	CCTCGCTTAGTGCTCCCTG	
7	CTTTATCCAATGTGCTCCTC	486
	TCTCCCTTCCCTGATTACCT	
8	TGGAGAAGCTCCCAACCAA	231
	TTCCCAAACACTCAGTGAAACA	
9	GTGGCACCATCTCACAATT	451
	ATGCTCCAGGCTCACCAAG	
10	TTCGCCAGCCATAAGTCCT	500
	TCATTCACTGTCCCAGCAAG	

Table S3. Clinical information and CTC counts for patients with non-small cell lung cancer.

Patient number	Age	Gender	CTC Count (per 5 ml)	Metastasis	Histology [a)]
LU1	46	Female	5	Yes	LUAD
LU2	44	Male	7	Yes	LUAD
LU3	47	Male	17	Yes	LUAD
LU4	44	Female	2	Yes	LUAD
LU5	62	Male	3	Yes	LUAD
LU6	49	Male	0	Yes	LUSC
LU7	62	Male	2	Yes	LUAD
LU8	78	Female	0	Yes	LUAD
LU9	85	Male	1	Yes	LUAD
LU10	60	Male	1	Yes	LUAD
LU11	73	Male	0	Yes	LUSC
LU12	73	Male	1	Yes	LUAD
LU13	64	Male	0	Yes	LUSC
LU14	60	Male	1	Yes	LUAD
LU15	64	Male	2	Yes	LUAD
LU16	63	Female	2	Yes	LUAD
LU17	36	Female	2	Yes	LUAD
LU18	64	Female	12	Yes	LUAD
LU19	65	Male	0	Yes	LUAD
LU20	45	Male	57	Yes	LUAD
LU21	55	Male	0	Yes	LUAD
LU22	59	Female	0	Yes	LUAD
LU23	51	Male	10	Yes	LUAD
LU24	41	Male	11	Yes	LUAD
LU25	46	Female	2	Yes	LUAD
LU26	63	Male	2	Yes	LUSC
LU27	85	Female	0	Yes	LUAD
LU28	57	Female	5	Yes	LUAD
LU29	61	Male	4	Yes	LUAD
LU30	30	Male	2	Yes	LUAD
LU31	67	Female	5	Yes	LUAD
LU32	61	Male	5	Yes	LUAD
LU33	50	Male	4	Yes	LUAD
LU34	63	Male	1	Yes	LUSC
LU35	73	Female	6	Yes	LUAD
LU36	38	Male	3	Yes	LUAD
LU37	45	Female	4	Yes	LUAD
LU38	78	Male	13	Yes	LUAD
LU39	51	Female	7	Yes	LUAD
LU40	54	Male	8	Yes	LUAD
LU41	72	Female	7	Yes	LUAD
LU42	63	Male	0	Yes	LUAD
LU43	65	Male	6	Yes	LUAD
LU44	49	Male	4	Yes	LUAD
LU45	52	Male	9	Yes	LUAD
LU46	54	Male	5	Yes	LUAD
LU47	65	Male	7	Yes	LUAD

LU48	65	Male	4	Yes	LUAD
LU49	54	Male	2	Yes	LUAD
LU50	58	Female	8	Yes	LUAD
LU51	53	Female	0	Yes	LUAD
LU52	51	Male	2	Yes	LUAD
LU53	55	Male	0	Yes	LUAD
LU54	57	Female	0	Yes	LUAD
LU55	54	Male	2	Yes	LUAD
LU56	65	Male	4	Yes	LUSC
LU57	63	Male	1	Yes	LUAD
LU58	39	Female	5	Yes	LUAD
LU59	86	Male	2	Yes	LUAD
LU60	75	Male	4	Yes	LUSC
LU61	78	Male	0	Yes	LUAD
LU62	58	Male	1	Yes	LUAD
LU63	52	Female	0	Yes	LUAD
LU64	65	Male	2	Yes	LUAD
LU65	66	Male	0	Yes	LUAD
LU66	72	Female	1	Yes	LUAD
LU67	64	Male	2	Yes	LUAD
LU68	67	Male	1	Yes	LUSC

[a] LUAD, lung adenocarcinoma; LUSC, lung squamous cell carcinoma.

Table S4. Clinical information and CTC counts for patients with liver cancer.

Patient number	Age	Gender	CTC Count (per 5 ml)	Metastasis	BCLC staging [a)]	AFP (ng/ml)
L1	51	Male	2	No	A	1250.2
L2	56	Male	3	Yes	B	99.1
L3	50	Male	4	Yes	C	721.7
L4	44	Male	1	No	A	711.8
L5	44	Male	0	No	A	1001.7
L6	58	Male	0	No	A	0.2
L7	41	Male	0	No	A	886.8
L8	31	Male	0	Yes	C	16.8
L9	53	Male	8	No	A	1.8
L10	47	Male	1	Yes	B	77.5
L11	54	Male	0	No	A	602.1
L12	38	Male	0	Yes	C	1.2
L13	59	Male	0	No	A	5.0
L14	62	Female	0	No	A	147.2
L15	54	Male	1	Yes	C	21.2
L16	65	Male	0	Yes	C	98.3
L17	64	Female	2	No	B	8.8
L18	45	Female	1	No	B	7.4
L19	62	Male	0	No	A	78.2
L20	48	Male	0	No	B	33.6
L21	53	Male	1	Yes	C	107.1
L22	55	Male	2	Yes	C	220.2

[a)] BCLC stageing, Barcelona Clinic Liver Cancer Staging.

Table S5. Clinical information and counts of TPN+ cells in pleural effusion from patients with lung cancer or benign diseases.

Number	Age	Gender	TPN+ cell Count (per 1 ml)	Diagnosis [a)]	Metastasis
PE1	64	Male	>1000	LUAD	Yes
PE2	73	Male	>1000	LUAD	Yes
PE3	62	Male	0	IAI	/
PE4	57	Male	0	DN	/
PE5	56	Male	0	CHF	/

[a)] LUAD, lung adenocarcinoma; IAI: intra-abdominal infection; DN: diabetic nephropathy; CHF: congestive heart failure.

Table S6. Datasets selected from TCGA database.

Type	Dataset
Normal tissue	55.6968.11A.01R.1949.07
	91.6829.11A.01R.1858.07
	44.2668.11A.01R.1758.07
	38.4632.11A.01R.1755.07
	50.5930.11A.01R.1955.07
	50.5933.11A.01R.1955.07
	55.6975.11A.01R.1949.07
	50.5932.11A.01R.1955.07
	55.6975.11A.01R.1949.07
	50.5939.11A.01R.1928.07
	55.6972.11A.01R.1949.07
	73.4676.11A.01R.1955.07
Lung cancer tissue	L9.A50W.01A.12R.A39D.07
	73.4675.01A.01R.1206.07
	49.4486.01A.01R.1206.07
	55.6972.01A.11R.1949.07
	62.A46P.01A.11R.A24H.07
	50.5932.01A.11R.1755.07
	73.4677.01A.01R.1206.07
	62.A62S.01A.11R.A24H.07
	55.8512.01A.11R.2403.07
	97.817401A.11R.2287.07
	55.7284.01B.11R.2241.07
	55.7227.01A.11R.2039.07
	93.A4JO.01A.21R.A24.07
	55.8092.01A.11R.2241.07
	50.5930.01A.11R.1755.07
	MP.A4TE.01A.22R.A466.07
	55.A490.01A.11R.A466.07
	50.7109.01A.11R.2039.07
	86.8359.01A.11R.2326.07
	55.8090.01A.11R.2241.07
	MP.A4SY.01A.21R.A24X.07

	49.AAR4.01A.12R.A41B.07
	55.7907.01A.11R.2170.07
	MP.A4TC.01A.11R.A24X.07
	86.8672.01A.21R.2403.07
	49.4494.01A.01R.1206.07
	38.4629.01A.02R.1206.07
	91.6829.01A.21R.1858.07
	86.8055.01A.11R.2241.07
	86.6562.01A.11R.1755.07
	73.4659.01A.01R.1206.07
	55.1592.01A.01R.0946.07
	50.5939.01A.11R.1628.07
	62.8398.01A.11R.2326.07
	50.5072.01A.21R.1858.07
	73.A9RS.01A.11R.A41B.07
	49.AAR9.01A.21R. A41B.07
	55.8620.01A.11R.2403.07
	86.A4D0.01A.11R.A24H.07
	38.4632.01A.01R.1755.07
	73.4676.01A.01R. 1755.07
	55.A4DF.01A.11R.A24H.07
	55.6968.01A.11R.1949.07
	44.7669.01A.21R.2066.07
	95.7562.01A.11R.2241.07
	86.A4JF.01A.11R.A24X.07
	64.5775.01A.01R.1628.07
	44.2668.01A.01R.A278.07
	44.2668.01A.01R.0946.07
	55.8089.01A.11R.2241.07
	55.6978.01A.01R.1949.07
	50.5933.01A.01R.1755.07
	55.6975.01A.01R.1949.07
	MP.A4T1.01A.21R.A24X.07
	44.2668.01B.02R.A277.07

References

[1] H. Shi, N. Zhao, D. Ding, J. Liang, B. Z. Tang, B. Liu, *Org. Biomol. Chem.* **2013**, *11*, 7289.

[2] S. Picelli, A. K. Bjorklund, O. R. Faridani, S. Sagasser, G. Winberg, R. Sandberg, *Nat. Methods* **2013**, *10*, 1096.
